# Call of the Void Scale: scale development, validation and psychometric properties

**DOI:** 10.3389/fpsyt.2025.1685791

**Published:** 2025-12-02

**Authors:** Lara Wiesmann, Laura Melzer, Tobias Teismann

**Affiliations:** 1 Mental Health Research and Treatment Center, Ruhr-Universität Bochum, Bochum, Germany; 2 Department of Clinical Psychological Intervention, University of Wuppertal, Wuppertal, Germany; 3 German Center for Mental Health, DZPG, partner site Bochum-Marburg, Bochum, Germany

**Keywords:** Call of the Void, high place phenomenon, scale development, factor analysis, intrusion

## Abstract

**Background:**

The inexplicable thought of jumping from a bridge, driving into oncoming traffic or walking in front of an oncoming train is known as the Call of the Void (COV) phenomenon. Previous studies have only focused on one COV aspect: the high place phenomenon (HPP), that is a sudden urge to jump when in a high place. In this study, we aimed to develop and validate the Call of the Void Scale (COVS) to measure the prevalence of a variety of these thoughts, their ego-dystonic character and associated safety behaviors.

**Methods:**

The study sample comprised *N* = 476 participants (71% female, mean age = 30.85 years, standard deviation = 11.5; age range: 18–67 years). Individuals completed the COVS between August 2023 and February 2024 in an anonymous online survey. We analyzed the scale´s factor structure, reliability, and validity through exploratory and confirmatory factor analyses. Bivariate correlations with existing measures of depression, stress, anxiety and suicidal ideation and behavior were calculated to assess convergent validity.

**Results:**

Using exploratory factor analysis on half of the sample (*n_1_* = 238), we derived a five-factor structure, which was interpreted as representing the frequency of COV in different situations (“frequency HPP”, “frequency car”, “frequency train”), the ego-dystonic character of the thoughts (“ego-dystonia”), and behavior to suppress or manage the thoughts (“safety behavior”). Confirmatory factor analysis on the other half of the sample (*n*_2_ = 238) showed a good fit for the five-factor solution. The subscales frequency HPP, frequency car, frequency train and safety behavior were positively correlated with depression, stress, anxiety and suicidal ideation and behavior. The ego-dystonia subscale was only correlated with the safety behavior subscale.

**Conclusion:**

The COVS seems to be a valid tool for the assessment of the Call of the Void phenomenon, measuring the prevalence of these thoughts, their ego-dystonic character and associated safety behaviors. The limitations of this study and implications for future research are discussed.

## Background

The sudden urge to jump when standing on a high place can be a frightening experience. It is known as the High Place Phenomenon (HPP, 1) and associated with the term Call of the Void or the French expression “L’Appel du Vide”. Previous studies have shown that HPP is a common experience, reported by 43-60% of participants across various samples (1–3). It is associated with suicidal ideation as well as depression, neuroticism and low self-esteem, suggesting a link to psychological vulnerability (1–3). The High Place Phenomenon (HPP) might be understood as a specific type of an unwanted intrusive thought (2). These are unpleasant, fleeting, and ego-dystonic thoughts or images, involving sexual, violent, or blasphemous content, often associated with anxiety or distress (4). In line with this comparison, the Call of the Void phenomenon is often described as a sudden, unwanted experience that evokes distress or confusion (5), which are hallmarks of intrusive cognitions (6).

To investigate the prevalence of the HPP and its relations to suicidality, Hames et al. (1) created the High Place Phenomenon Index (HPPI). The questionnaire consists of the three items (Item1: “When standing on the edge of a tall building or walking on a bridge, have you ever had the urge to jump? How often has this happened in your lifetime?”, Item 2: “When you see a tall building or are walking on a bridge, have you ever thought about what it would be like to jump off of it? How often has this happened in your lifetime?”, and Item 3: “When you are inside a tall building, have you ever imagined jumping out a window? How often has this happened in your lifetime?”*)* rated on a 6-point Likert type scale ranging from “1=never” to “6 = always”. The scale demonstrated good internal consistency (Cronbach’s α = .85) in a sample of 432 undergraduate college students from the United States. Confirmatory factor analysis supported a one-factor solution, with all items loading significantly on the factor (λ = .76–.84; *p* <.001 for all). The HPP was shown to correlate with anxiety sensitivity, depression and suicidal ideation. Based on these findings, Teismann et al. (2) used a German translation of the HPPI that was developed by means of a translation-back-translation procedure according to relevant guidelines for the translation of psychometric instruments (7). They investigated the cross-cultural prevalences of the high place phenomenon and its clinical relevance by using one German online sample (*N* = 276) and one outpatient sample (*N* = 94). Internal consistencies were good in both samples (online sample α = .84; patient sample α = .86). Suicidal ideation as well as anxiety sensitivity, depression and anxiety were positively associated with the HPP in the online sample whereas depression, anxiety and suicidal ideation were unrelated to experiences with the HPP in the patient sample. Teismann et al. (2) compared the group of lifetime and current suicidal ideators to non-ideators and found significant higher HPPI-scores in the ideator groups. There were significant correlations between the HPP and depression, anxiety and anxiety sensitivity in both groups of the online sample.

The German HPPI was also used in an even larger sample of *N* = 612 anxiety patients (suffering from fear of flying) to investigate the correlations of the phenomenon with personality factors and positive and negative health markers (3). Consistent with the literature, the scale showed good internal consistency (*α* = .79; 3). In this study, openness to experiences, neuroticism and suicidal ideation were positively related to the high place phenomenon, whereas agreeableness, self-efficacy and self-esteem were negatively related.

The existing research has focused exclusively on the urge to jump from high places. However, it is important to note that online communities often discuss the phenomenon more broadly. There, the term “Call of the Void” is used to describe a broader range of experiences, including the HPP, as well as the urge to drive into oncoming traffic or to step in front of a train (5, 8–10). Despite their apparent similarity to the HPP, these experiences have not yet been systematically investigated. Therefore, we aimed to develop the Call of the Void Scale.

The questionnaire is inspired by the High Place Phenomenon Index (1), yet, it is expanded to assess a broader range of experiences. As such, the scale is designed to assess the frequency of the Call of the Void Phenomenon in three situations: being at heights, driving a car and standing at a train platform. These three contexts were the most frequently discussed (5, 8–10) and are characterized by a sudden, intrusive impulse to perform potentially fatal actions. During item development, other contexts that were occasionally mentioned online, such as handling sharp objects or jumping into water, were excluded due to their lower significance and presence in the online discourse.

To account for possible parallels between COV and obsessive-compulsive thoughts and to compare their underlying mechanisms, additional items were developed: Two items “How alien is this idea to your personality?” and “How exaggerated do you find this thought?” address the ego-dystonic nature of COV thoughts. These items were developed based on the idea that ego-dystonia is the main difference between obsessions and other types of thoughts and worries (11–16).

In line with the conceptual comparison of Call of the Void experiences to obsessive-compulsive thought patterns, we added further items to measure safety behaviors. Safety behaviors are actions aimed at preventing or minimizing a feared outcome (17). These actions seem useful and provide short-term anxiety relief, but they are one of the key factors in maintaining these fearful thoughts (6, 18). To analyze the possible role of safety behaviors in COV-related thoughts, we included the following items in the questionnaire: “Do you try to get rid of the thought by ignoring or suppressing it?”, “Do you try to make the thought harmless through other thoughts or actions?” and “Do you do anything to ensure that you do not act on the thought?”.

In summary, the present study represents an initial exploratory step in the development and validation of the Call of the Void Scale. It aims to expand the conceptual and empirical understanding of the Call of the Void by developing a self-report questionnaire that includes not only the high place phenomenon, but also COV- related thoughts while driving a car or standing at a train platform. It assesses the frequency of these experiences, the ego-dystonic character of these thoughts and safety behaviors that might be engaged. The objective of this study is to develop a reliable and valid instrument with which to measure these experiences. To ensure psychometric soundness, we examined the quality of the questionnaire items, its internal consistency, and its underlying structure. Exploratory and confirmatory factor analyses were used to identify and validate a coherent factor structure. Theoretically relevant variables were used to assess validity. Based on previous findings with the HPPI, we expected positive correlations with depression and suicidal ideation (1–3).

## Methods

### Participants

The sample comprises of *N* = 476 participants (71% female, 28% male, 1% diverse; *M*_age_=30.85, *SD*_age_=11,5, range: 18–67 years). Most participants (85.1%) were German citizens, while 14.3% held other nationalities (9.0% Austrian, 0.4% Turkish, 0.2% Polish). In terms of employment status, 47.7% individuals were employees, 46.3% students or trainees, 0.6% job seekers, 1.3% housewives or househusbands, 1.31% were retired, and 3.2% had another job. Among the participants, 9.9% of participants were currently in treatment, 20.6% reported past treatment. The most frequently reported mental health diagnoses were depressive disorders (5.3% current, 7.4% past) and anxiety disorders (2.1% current, 2.5% past), while other diagnoses, such as trauma-related, personality, or eating disorders were less common (current and past ranging from 0.2-1.3%). A total of 67.2% of participants reported having no prior or current experience of treatment. Two-hundred seventeen participants (45.6%) reported some suicidal ideation in the last 4 weeks (SIBS score > 1) and sixty-five participants (13.7%) indicated that they had attempted suicide at least once in their life (range 1 to 10+ with *M* = 1.97 suicide attempts).

Participants completed the measures included in this study in an anonymous online survey between August 2023 and February 2024 using the So-Sci-server (https://www.soscisurvey.de/). Recruitment took place via advertisements at local universities and was open to individuals regardless of previous experiences with the Call of the Void Phenomenon. In addition, an article published on the Ruhr University news portal about the COV invited individuals to participate. To take part in the study, they had to give their consent to participation at the beginning of the study and had to be at least 18 years old. No exclusion criteria were applied concerning mental disorders or suicidal ideation. Participation in the study was not remunerated. Students received course credit for their participation. The contact information of the principal investigator was available to all participants if they had any questions or concerns. Furthermore, contact addresses for support services in an event of acute psychological crisis were provided. The study was approved by the Ethics Committee of the Ruhr-Universität Bochum (Ethics vote 873).

### Measures

Call of the Void Scale (COVS). We developed the COVS, a 27-item self-report questionnaire designed to assess the Call of the Void in three situations: being at heights (“When standing on the edge of a tall building or walking on a bridge, have you ever had the thought about jumping down?”), driving a car (“When you drive your car, have you ever had the thought of hitting a tree or driving into the oncoming lane?”) and standing at a train platform (“When you stand at a train platform, have you ever had the thought of walking in front of a train?”). The scale uses a 5-point Likert Scale ranging from “1= never” to “5= always” to assess the frequency of these thoughts (“How often has this happened in your life?”, “How often has it happened in the last month?”). In addition, the questionnaire asks about the ego-dystonic quality of these experiences (“How alien is this idea to your personality?”, “How exaggerated do you find this thought?”) and possible safety behaviors to manage or suppress these experiences (“Do you try to get rid of the thought by ignoring or suppressing it?”, “Do you try to make the thought harmless through other thoughts or actions?” and “Do you do anything to ensure that you do not act on the thought?”). All items are rated on a 5-point Likert Scale, ranging from “1=not at all” to “5=very much”.

Suicide Ideation and Behavior Scale (SIBS, 19). The SIBS assesses the frequency of suicidal ideation in the past four weeks with six items (e.g., “During the past four weeks, … I thought it would be better if I wasn’t alive, … I have seriously considered killing myself”). The items are rated on a 6-point Likert scale ranging from “1=never” to “5 = many times every day”. Higher scores indicate greater severity of suicidal ideation. The occurrence/frequency of suicide attempts within the last 4 weeks as well as lifetime suicide attempts are assessed with three further SIBS-items (“In the course of my life/in the last 4 weeks. I have tried to kill myself - and I really wanted to die”, “How many times have you tried to kill yourself?”). The scale has been found to have strong internal consistency in the validation sample (Cronbach’s α ≥.92; 19), as well as in the current sample (α = .85).

**Depression Anxiety Stress Scales** (DASS; 20). The DASS assesses depressive mood, anxiety, and stress during the past week. It consists of the three subscales Stress (“I found myself getting upset rather easily”), Depression (“I felt that I had nothing to look forward to”) and Anxiety (“I was aware of dryness in my mouth”). The 21 items are rated on a 4-point (0-3) Likert scale. Internal consistency was good in total ranging from *α* = 0.88 – 0.96 (21). Internal consistency of the DASS and its subscales were good in this sample as well (DASS: *α* = 0.93, Subscale Depression α = .92, Subscale Anxiety α= .80, Subscale Stress α = .87).

### Statistical analyses

Statistical analyses were conducted using R Studio (version: 2025.05.0 + 496; 22). To ensure data quality, 45 participants were excluded due to missing data or incomplete questionnaire sections, resulting in a final sample of 476 participants.

Descriptive data was collected, and the distribution of the data was checked using the Kolmogorov-Smirnov test. In addition, means, standard deviation, skewness and kurtosis of the items were analyzed.

The dataset was randomly split in half prior to conducting both the exploratory factor analysis (EFA, *n_1_* = 238) and the confirmatory factor analysis (CFA, *n_2_* = 238). The appropriate number of factors was determined using parallel analysis (*psych* package; 23), and an assessment of the model quality. In the EFA, we examined the dimensionality of the scale and the loadings of the items by conducting a maximum likelihood factor analysis with oblimin rotation. We tested the factor structure identified in the EFA by using the second half of the sample in a confirmatory factor analysis (CFA, *lavaan* package; 24). The Kaiser-Meyer-Olkin (KMO) measure of sampling adequacy (25) and Bartlett’s test of sphericity (26) were used to test the data’s suitability for confirmatory factor analysis. To evaluate the fit of the model, we used global and local fit indices. The Chi-square test determines whether there is a significant difference between the model and the data. Therefore, a non-significant result suggests that the model fits the data well. However, the test is oversensitive in large samples (27). The RMSEA (Root Mean Square Error of Approximation) measures how well the prior model reproduces the sample data while accounting for model complexity; values between.05 and.08 indicate a good fit, and values up to.10 are still acceptable (28, 29). The CFI (Comparative Fit Index) and the TLI (Tucker-Lewis Index) measure the improvement of fit of the specified model in comparison to the restricted baseline model (29). They indicate good fit to acceptable fit when > 0.95 to > 0.90. The SRMR (standardized root mean square residual) measures the average difference between observed and predicted covariances. Values below 0.08 indicate a good fit and below 0.11 acceptable fit (29). As local fit indices, factor loadings above 0.40 were considered as good (30). The internal consistency of the scale was determined by calculating Cronbach’s α, with values ≥0.70 indicating good internal consistency (31). Convergent validity was evaluated by calculating Pearson correlation coefficients between the COVS, depression, stress and anxiety (DASS), and suicidal ideation (SIBS). Furthermore, correlation analyses were conducted separately for suicide ideators and non-ideators.

## Results

### Item analysis

The whole sample (*N* = 476) was used for the primary item analysis. The Kolmogorov-Smirnov test (*p* = .388) indicated that the data were normally distributed. Item-total correlations ranged from *r* = .29 to *r* = .71. Most items showed item correlations above *r* = .40, which is considered satisfactory. Only one item (HPP: “How alien to your personality is this thought?”) had a correlation of *r* = .29, which is slightly below the typical threshold of.30.

### Parallel analysis and exploratory factor analysis

The significance of Bartlett’s test of sphericity (26) indicates sufficient correlations among the items (χ²(26) = 487.35, *p* <.001). In addition, the Kaiser–Meyer–Olkin (KMO; 25) measure of sampling adequacy confirmed that the data were suitable for exploratory factor analysis (KMO = .83). Parallel analysis based on *n*_1_ = 238 with all 27 items suggested seven factors. However, by inspecting the model, three items showed only weak factor loadings (<.50) as well as cross-loadings in two factors (three items; >.30) or low communalities (one item, <.40). Therefore, five items and their counterparts (four items) referring to the same item in the other situations (“Do you try to get rid of the thought by ignoring or suppressing it?”, “Do you try to make the thought harmless through other thoughts or actions?”) were omitted.

A five-factor structure emerged in the second parallel analysis based on the remaining 18 items. They were interpreted as representing “frequency HPP”, “frequency Car”, “frequency Train”, “ego-dystonia” and “safety behavior”, explaining 72% of the total variance.

The remaining items loaded significantly on their factors without substantial cross-loadings (>.30), all communalities exceeded.48, and the average item complexity was low (1.2–1.3).

### Confirmatory factor analysis

The five-factor model of the EFAs was investigated by confirmatory factor analysis with maximum likelihood estimation on the second half of the sample (*n_2_* = 238). The initial CFA, which included the remaining 18 items, showed an insufficient fit: *χ*^2^(125) = 661.366, (*p* = .000), TLI = 0.842, CFI = 0.807, RMSEA = 0.134 [90% CI 0.123 to 0.143]. Only SRMR = .082 supported a good model fit. Despite the acceptable SRMR and strong factor loadings, the model did not fit well overall. Based on these results three items were removed to improve the model fit and enhance conceptual clarity and parsimony (32, 33): The item “How exaggerated do you find this thought?” in the context of HPP as well as in the context of the thought of walking in front of a train, showed a combination of weak factor loadings (= .59), low communalities (h= .43), and high complexity (com=1.4). Due to the coherence of content and form of the questionnaire, the identical item in the context of the thought of hitting a tree of driving into the oncoming lane (“How exaggerated do you find this thought?”) was also removed. The final version with 15 items and 5 factors is depicted with all item characteristics in **Table 1**.

**Table 1:** Item characteristics descriptive statistics, and standardized factor loadings from the exploratory factor analysis (EFA) of all 15 items of the final COVS.

Item number	Situation	Factor	Item	Descriptive statistics							Exploratory factor analysis: Factor loadings
				min	max	*m*	*SD*	skew	Kurt.	SE	
1	High Place Phenomenon (HPP	Frequency HPP	When you stand on the edge of a tall building or walk across a bridge, have you ever had the thought of jumping down?	1	5	2.69	1.11	0.05	-0.89	0.05	0.95
2		Frequency HPP	How often has this happened in your life?	1	5	2.62	1.09	0.05	-1.0	0.05	0.91
3		Frequency HPP	How often has it happened in the last month?	1	5	1.72	0.99	1.22	0.51	0.05	0.48
4		Ego-dystonia	How alien to your personality is this thought?	1	5	2.86	1.44	0.03	-1.39	0.07	0.71
5		Safety behavior	Do you do anything to ensure that you do not act on the thought?	1	5	2.35	1.53	0.64	-1.16	0.07	0.79
6	Car	Frequency Car	When you drive your car, have you ever had the thought of hitting a tree of driving into the oncoming lane?	1	5	2.58	1.16	0.01	-1.16	0.05	0.94
7		Frequency Car	How often has this happened in your life?	1	5	2.41	1.08	0.16	-1.18	0.05	0.93
8		Frequency Car	How often has it happened in the last month?	1	5	1.67	0.94	1.25	0.59	0.04	0.65
9		Ego-dystonia	How alien to your personality is this thought?	1	5	3.09	1.53	-0.12	-1.46	0.07	0.79
10		Safety behavior	Do you do anything to ensure that you do not act on the thought?	1	5	2.23	1.53	0.8	-0.96	0.07	0.79
11	Train	Frequency Train	When you stand at a train platform, have you ever had the thought of walking in front of a train?	1	5	2.44	1.23	0.29	-1.12	0.06	0.97
12		Frequency Train	How often has this happened in your life?	1	5	2.26	1.13	0.43	-0.99	0.05	0.92
13		Frequency Train	How often has it happened in the last month?	1	5	1.61	0.97	1.57	1.59	0.04	0.69
14		Ego-dystonia	How alien to your personality is this thought?	1	5	2.94	1.6	0.02	-1.57	0.07	0.79
15		Safety behavior	Do you do anything to ensure that you do not act on the thought?	1	5	2.19	1.52	0.84	-0.89	0.07	0.88

COVS, Call of the Void Scale; HPP, High Place Phenomenon, descriptive statistics (minimum, maximum; *m*, Mean; *SD*, Standard Deviation; Kurt, kurtosis; SE, standard error).

The final version with 15 items and 5 factors demonstrated reasonable to good model fit with CFI = .938, TLI = .919 and SRMR = .061. The RMSEA = .092 [90% CI 0.079 to 0.105] was slightly higher but still acceptable. Only the overly sensitive Chi-square test indicated a difference between the model and the data (*χ*^2^ = 241.190, *df* = 80, *p* = .000).

**Figure 1:**
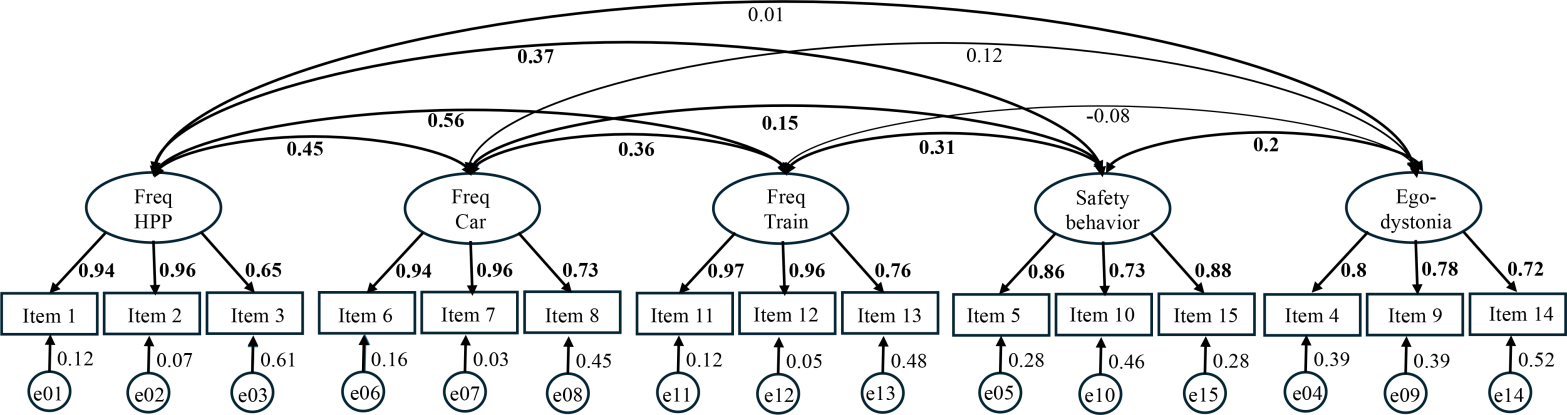
Completely standardized five-factor solution of the confirmatory factor analysis (CFA; *n* = 238). Ellipses represent latent variables, and rectangles represent observed items. Double-headed arrows between latent variables represent the intercorrelations among the five factors, with numerical values corresponding to the standardized correlation coefficients (r) and thicker lines indicating stronger standardized loadings. The numerical values on the single-headed arrows indicate the completely standardized factor loadings. Circles labeled e01–e14 denote the residual variances (error terms) of the observed items. Freq HPP = Frequency High Place Phenomenon; Freq Car = Frequency Car; Freq Train = Frequency Train.

All standardized factor loadings were significant. They ranged from.624 to.985 (see **Figure 1**). These results provide evidence for a five-factor model, which is theoretically and empirically supported.

### Reliability

Cronbach´s alpha was calculated as a measure of internal consistency with scores > 0.70 considered as good (31). The subscales demonstrate good internal consistencies: frequency Train α = 0.91, frequency Car α = 0.90, frequency HPP α = 0.87, safety behavior α = 0.86 and ego-dystonia α = 0.80.

### Validity

To determine convergent validity, correlations were calculated between the different subscales of the final COVS model and existing measures, including the DASS subscales and the SIBS. Correlations >0.30 were considered as moderate and correlations >0.50 were considered as strong (34). As expected, the three frequency subscales had significant and low to moderate correlations with depression (DASS-D), anxiety (DASS-A), stress (DASS-S) and suicidal ideation (SIBS). The subscale safety signals showed significant but low correlations with the other constructs. Only the subscale ego-dystonia showed no significant correlations to depression, anxiety, stress or suicidal ideation (see **Table 2**).

**Table 2:** Correlations between the five COVS scales and related constructs.

Construct	Scale	*m*	*SD*	1	2	3	4	5	6	7	8	9
1	COVS: Frequency Train	2.11	1.03	1	**.626**	**.571**	**.270**	.010	**.344**	**.273**	**.234**	**.423**
2	COVS: Frequency Car	2.20	.87		1	**.512**	**.262**	.042	**.353**	**.305**	**.240**	**.350**
3	COVS: Frequency HPP	2.34	.95			1	**.307**	-.036	**.331**	**.194**	**.210**	**.340**
4	COVS: Safety behavior	2.26	1.35				1	**.296**	**.146**	**.183**	.119	**.172**
5	COVS: Ego-dystonia	2.43	.96					1	-.063	.036	-.023	-.100
6	DASS-D	1.82	.76						1	**.637**	**.694**	**.623**
7	DASS-A	1.48	.51							1	**.586**	**.446**
8	DASS-S	2.13	.71								1	**.347**
9	SIBS	1.25	.48									1

*m*, Mean; *SD*, Standard Deviation, correlations in bold letters were significant at *p <.05*. COVS, Call of the Void Scale; DASS, Depression Anxiety Stress Scales: DASS-D Subscale Depression, DASS-A Subscale Anxiety, DASS-S: Subscale Stress (20), SIBS, Suicide Ideation and Behavior Scale (19).

**Table 3** presents the correlation analyses für suicide ideators and non-ideators, analyzed separately. To compare the mean scores between both groups, independent-samples t-tests were used. The group of suicidal ideators showed significantly higher scores on all variables except for ego-dystonia (all *p* <.001). In that regard, the non-ideators reported significantly higher rates (*p* = .002).

**Table 3:** Correlations between the five COVS scales and related constructs in suicidal ideators and non-ideators.

Construct	Variable	*m*	*SD*	1	2	3	4	5	6	7	8	9
1a- 8a Suicidal ideators (n=217) and 1b-8b Non-ideators (n=259)												
1a	COVS: Frequency Train	2.51	1.05	1	**0.59**	**0.49**	**0.18**	-0.02	**0.27**	**0.23**	**0.13**	**0.38**
1b	COVS: Frequency Train	1.77	0.87	1	**0.59**	**0.54**	**0.27**	**0.13**	0.05	0.09	0.10	
2a	COVS: Frequency Car	2.46	0.89		1	**0.40**	**0.19**	0.00	**0.32**	**0.25**	**0.16**	**0.34**
2b	COVS: Frequency Car	1.98	0.78		1	**0.53**	**0.26**	**0.14**	**0.16**	**0.23**	**0.16**	
3a	COVS: Frequency HPP	2.69	0.92			1	**0.18**	**-0.14**	**0.24**	0.08	0.08	**0.28**
3b	COVS: Frequency HPP	2.05	0.88			1	**0.35**	0.11	**0.13**	0.11	**0.12**	
4a	COVS: Safety behavior	2.52	1.33				1	**0.23**	0.06	**0.14**	0.07	0.13
4b	COVS: Safety behavior	2.04	1.32				1	**0.39**	0.06	**0.13**	0.05	
5a	COVS: Ego-dystonia	2.30	0.88					1	-0.06	**0.13**	0.04	-0.06
5b	COVS: Ego-dystonia	2.53	1.01					1	0.08	0.03	0.00	
6a	DASS-D	2.27	0.79						1	**0.54**	**0.63**	**0.54**
6b	DASS-D	1.44	0.47						1	**0.66**	**0.61**	
7a	DASS-A	1.67	0.59							1	**0.51**	**0.40**
7b	DASS-A	1.32	0.36							1	**0.57**	
8a	DASS-S	2.41	0.73								1	**0.24**
8b	DASS-S	1.89	0.59								1	

*m*, Mean; *SD*, Standard Deviation, correlations in bold letters were significant at *p <.05*. COVS, Call of the Void Scale; DASS, Depression Anxiety Stress Scales; DASS-D Subscale Depression; DASS-A Subscale Anxiety, DASS-S; Subscale Stress (20), SIBS, Suicide Ideation and Behavior Scale (19).

Among ideators, depression and safety behavior were the only variables significantly correlated to all three types of COV experiences (train, car, HPP). Anxiety and stress were positively correlated with both Train and Car COV. Ego-dystonia showed a small negative association with the High Place Phenomenon. In the non-ideator group, the pattern differed notably. Ego-dystonia was positively associated with Train and Car COV and safety behavior correlated significantly with all kinds of COV experiences. Depression and Stress showed small associations with COV Car and HPP and only a weak only weak, nonsignificant correlations with COV Train in the non-ideator group. Anxiety was only associated with COV while driving a car. However, when tested statistically using Fisher´s r-to-z transformation, the differences in the correlations between the non-ideators and ideators were not significant following Benjamini-Hochberg (all *p* > 0.05) correction.

## Discussion

The aim of the present study was to develop an instrument to assess the Call of the Void (COV) phenomenon, i.e. the sudden urge to jump when standing on a high place, inspired by the High Place Phenomenon Index (HPPI; 1). In online communities (5, 8–10), the COV is not limited to the sudden urge to jump from high places, it also includes the urge to drive into oncoming traffic or step in front of a train. To account for possible parallels with obsessive-compulsive thoughts, the scale assesses not only the frequency of these thoughts, but also the ego-dystonic character and possible safety behaviors to suppress them (cf. DIPS; 35).

After conducting an initial exploratory factor analysis (EFA), nine items were removed to refine the model. The following confirmatory factor analysis (CFA) showed an insufficient fit, so that three additional items were removed. The final exploratory factor analysis of the remaining 15 items revealed a five-factor structure for the Call of the Void Scale (COVS), which was confirmed by confirmatory factor analysis (CFA). The five factors were labeled “frequency HPP”, “frequency train”, “frequency car”, “ego-dystonia” and “safety behavior”, with three items each. The final version includes nine items assessing the prevalence of the thoughts and three items each assessing “egodystonia” and “safety behavior”. This item distribution underscores the scales´ theoretical focus on capturing the occurrence of the different COV scenarios. The internal consistency of each subscale was good. The construct validity of the COVS was supported by the expected associations between the frequency subscales and measures that had previously shown associations with HPP, such as depression, anxiety, stress and suicidal ideation. In summary, the COVS appears to be a valid and reliable instrument for measuring the Call of the Void in the current sample.

When analyzed separately, we found a small negative correlation between the frequency of the thought of jumping from a high place and ego-dystonia and no significant associations between COV car and train and ego-dystonia in the suicidal ideator group.

In contrast, within the non-ideators group, we could find a significant positive correlation between ego-dystonia and the frequency of the Call of the Void phenomenon when driving a car and standing at a train platform. There was no such correlation between ego-dystonia and HPP. Furthermore, ego-dystonia was significantly associated with safety behavior, which itself was positively correlated with the frequency of COV across all situations. This pattern suggests a possible indirect pathway in which ego-dystonic appraisals may influence the COV frequency via safety behaviors, a mechanism warranting further investigation in future research. However, the group differences in the strength of the correlations did not remain after correcting for multiple comparison.

Nevertheless, the overall pattern of associations is aligns with theoretical models describing the development and maintenance of obsessive-compulsive thoughts: according to Rachmann (36–38, the ego-dystonic interpretation of intrusive thoughts can trigger a cognitive process wherein individuals question the origin and meaning of the thought (“Why would a person like me have a thought like this?”). This may lead to an overinterpretation of the thought’s meaning (“Perhaps, subconsciously, I really want to die”), resulting in emotional distress and the use of safety behaviors. Such behaviors can increase the frequency and salience of intrusive thoughts, leading individuals to assign them greater meaning or importance (39). Future research should examine the similarities and differences of these obsessive-compulsive thoughts and the Call of the Void phenomenon by investigating the relations to cognitive biases commonly associated with obsessive-compulsive disorder (OCD), such as thought-action fusion and perfectionism (40).

However, these cognitive-affective mechanisms do not appear to operate similar in the suicide ideator group. In this group, the thoughts of considering life-threatening actions seem to be perceived as congruent with the individual’s internal experiences and thus are less likely to be appraised as intrusive or alien. These findings align with the Interpersonal Psychological Theory of Suicide (IPTS; 41), which states that suicidality often arises as a response to psychological pain, from feelings of thwarted belongingness and perceived burdensomeness: individuals believe that others would be better off without them and that they are alone trapped in their suffering. Thus, the wish to die becomes ego-syntonic, reflecting the person´s conscious desire rather than intrusive impulse. In contrast, ego-dystonic suicidal ideation, conceptualized as *suicidal obsessions* (often in the context of OCD, 42), is typically experienced as unwanted and distressing and incongruent with the person´s values. These thoughts are considered to carry a lower risk of suicide attempts, as the distress and fear associated with these thoughts serve as a protective factor and distinguish them from true suicidality (43, 44).

Within the suicide ideators, significant association were observed between the frequency of COV and levels of depression, anxiety and suicidal ideation. These findings mirror the correlations between suicidal ideation and depression and anxiety symptoms, suggesting that in individuals with suicidal ideation, COV experiences may represent a distinct subtype or manifestation of suicidal thought processes.

Despite the promising psychometric properties of the Call of the Void Scale (COVS); the following limitations should be considered. The primary goal of this process was to identify a preliminary structure of the instrument, so analyses were conducted within a single sample and item refinement was based on conceptual and empirical consideration. Furthermore, the participants were recruited by means of a press appeal by the Ruhr University Bochum news portal. People who had experienced COV were specifically called upon and the press appeal drew the attention of people who had searched for the relevant keywords online. This resulted in a pre-sorting process. This, together with the sample consisting of mainly Caucasians, limits the generalizability of the results. Therefore, future studies should aim to recruit a more representative sample to ensure the applicability of the Call of the Void Scale to the general population. Moreover, future research should seek to replicate the present findings of the self-selected online sample in clinical populations, especially among individuals diagnosed with obsessive-compulsive disorders (OCD) or mood disorders, to assess whether the factor structure and correlates of the COVS hold in diagnostically relevant groups. Moreover, item selection and refinement during the EFA and CFA were conducted within the same dataset, which may have introduced overfitting. No test-retest reliability was measured. Therefore, the psychometric properties of the COVS as well as the underlying factor structure should be regarded as preliminary. Future studies should validate the final version of the questionnaire in an independent and representative sample. In addition, the final version of the COVS includes nine items assessing prevalence and three items each assessing ego-dystonia and safety behavior, which reflects the theoretical emphasis of the scale on the occurrence of the phenomena, but also results in a certain imbalance across subdomains.

Additionally, the cross-sectional design of this study limits the possibility to interpret the relationships between COV experiences, suicidal ideation, ego-dystonia and safety behaviors. Longitudinal designs are necessary to examine temporal dynamics and potential causal mechanisms, such as the hypothesized increase in COV thoughts over time due to ego-dystonic interpretations preceding the emergence of safety behaviors. Lastly, the interpretation of ego-dystonia and safety behaviors was guided by existing models of obsessive-compulsive thoughts (e.g., 6, 15, 17, 18), as it provided a useful conceptual framework. Nevertheless, the present study did not include direct measures of cognitive distortions like thought-action-fusion, responsible beliefs, or meta-cognitive appraisals (40). Including those constructs in future research would allow a more precise testing of transdiagnostic mechanism that may underlie the COV in both ideators and non-ideators. In sum, while the study provides an important foundation for the measurement of COV and highlights meaningful group differences between suicidal ideators and non-ideators, further research is needed to clarify the clinical relevance, underlying mechanism, and potential implications of the Call of the Void experiences.

Despite these limitations, the present study provides initial evidence suggesting that the COVS is a psychometrically sound measure of the Call of the Void beyond the high place phenomenon. Although it is not yet intended or validated for diagnostic use, the instrument can be used to expand the conceptual and empirical understanding of the High Place Phenomenon in its prevalences, ego-dystonia character and initiated safety behaviors and investigate the broader experiences of the Call of the Void while driving a car or standing at a train platform.

## Conclusion

The COVS seems to be a valid assessment tool for the Call of the Void phenomenon. It captures thoughts of jumping from a bridge, driving into oncoming traffic, or walking in front of an oncoming train. The COVS assesses the prevalence and ego-dystonic character of these thoughts, as well as associated safety behaviors and highlights differences between suicidal ideators and non-ideators. Therefore, it paves the way for further research on the potential nature of COV, helps avoid misinterpretations, and improves our understanding of the emotional perception and cognitive processing of danger in general.

## Data Availability

The raw data supporting the conclusions of this article will be made available by the authors, without undue reservation.
